# 2-(1,3-Dibenzyl­imidazolidin-2-yl­idene)malononitrile

**DOI:** 10.1107/S1600536808014025

**Published:** 2008-05-21

**Authors:** Xiao-Zhong Feng, Fu-Feng Yan, Zhen-Ping Li

**Affiliations:** a Henan Provincial Key Laboratory of Surface & Interface Science, Zhengzhou University of Light Industry, Zhengzhou 450002, People’s Republic of China; b Light Industry Vocational College, Zhengzhou University of Light Industry, Zhengzhou 450002, People’s Republic of China

## Abstract

In the title mol­ecule, C_20_H_18_N_4_, the imidazolidine ring makes dihedral angles of 86.74 (2) and 81.18 (3)° with the two phenyl rings. In the absence of classical inter­molecular inter­actions, the crystal packing is stabilized by van der Waals forces.

## Related literature

For the crystal structures of related compounds, see: Adhikesavalu & Venkatesan (1982 ▶). For details of the biological activities of imidazolidine-containing compounds, see: Sasho *et al.*, 1994 ▶. For bond-length data, see: Allen *et al.* (1987 ▶).
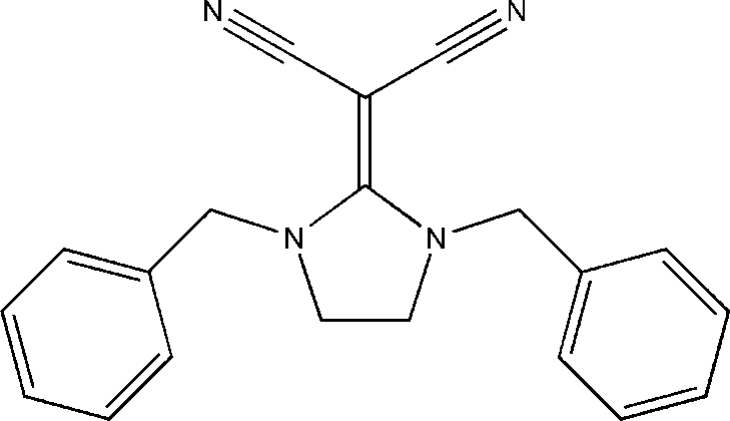


## Experimental

### 

#### Crystal data

C_20_H_18_N_4_
*M* *_r_* = 314.38
Orthorhombic, 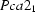

*a* = 15.445 (3) Å
*b* = 9.753 (2) Å
*c* = 11.411 (2) Å
*V* = 1718.9 (6) Å^3^
*Z* = 4
Mo *K*α radiation
μ = 0.07 mm^−1^
*T* = 293 (2) K
0.24 × 0.14 × 0.08 mm

#### Data collection

Rigaku R-AXIS RAPID IP area-detector diffractometer
Absorption correction: multi-scan (*ABSCOR*; Higashi, 1995 ▶) *T*_min_ = 0.982, *T*_max_ = 0.994
12952 measured reflections
1607 independent reflections
1291 reflections with *I* > 2σ(*I*)
*R*_int_ = 0.038

#### Refinement

*R*[*F* ^2^ > 2σ(*F* ^2^)] = 0.041
*wR*(*F* ^2^) = 0.114
*S* = 1.13
1607 reflections
218 parameters
1 restraint
H-atom parameters constrained
Δρ_max_ = 0.17 e Å^−3^
Δρ_min_ = −0.15 e Å^−3^

### 

Data collection: *RAPID-AUTO* (Rigaku, 2004 ▶); cell refinement: *RAPID-AUTO*; data reduction: *RAPID-AUTO*; program(s) used to solve structure: *SHELXTL* (Sheldrick, 2008 ▶); program(s) used to refine structure: *SHELXTL*; molecular graphics: *SHELXTL*; software used to prepare material for publication: *SHELXTL*.

## Supplementary Material

Crystal structure: contains datablocks I, global. DOI: 10.1107/S1600536808014025/hg2400sup1.cif

Structure factors: contains datablocks I. DOI: 10.1107/S1600536808014025/hg2400Isup2.hkl

Additional supplementary materials:  crystallographic information; 3D view; checkCIF report

## Figures and Tables

**Fig. 1.**
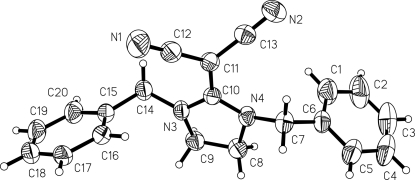
The molecular structure of (I), with atom labels and 40% probability displacement ellipsoids for non-H atoms.

## supplementary crystallographic information

### Comment

Imidazolidine is an important group in organic chemistry. Many compounds containing imidazolidine groups possess a broad spectrum of biological activities (Sasho *et al.*, 1994). Here, we report the crystal structure of (I).

In (I) (Fig. 1), all bond lengths are normal (Allen *et al.*, 1987) and in a good agreement with those reported previously (Adhikesavalu & Venkatesan., 1982). The imidazolidine ring (C8—C10/N3/N4) makes dihedral angles of 86.74 (2) and 81.18 (3)°, respectively, with two benzene rings (C1—C6; C15—C20). In the absence of classical intermolecular interactions, the crystal packing is stabilized by van der Waals forces.

### Experimental

A solution of 2-(imidazolidin-2-ylidene)malononitrile 1.34 g (10 mmol) and sodium hydride 0.3 g dissolved in anhydrous acetonitrile (20 ml), and dropwise added over a period of 10 min to a solution of 1-(chloromethyl)benzene 2.53 (20 mmol) in acetonitrile (10 ml) at 273 K. The mixture was stirred at 353 K for 3 h. The solvent was removed and the residue was purified by flash chromatography (1:1 cyclohexane:dichloromethane) to give I as a white solid (2.67 g, 85%). Single crystals suitable for X-ray measurements were obtained by recrystallization from ethanol at room temperature.

### Refinement

H atoms were positioned geometrically and refined using a riding model, with C—H = 0.93 or 0.97 Å, with *U*_iso_(H) = 1.2 times *U*_eq_(C). In the absence of significant anomalous scattering effects, Friedel pairs were merged.

### Crystal data

**Table:**

C_20_H_18_N_4_	*F*_000_ = 664
*M**_r_* = 314.38	*D*_x_ = 1.215 Mg m^−^^3^
Orthorhombic, *P**c**a*2_1_	Mo *K*α radiation λ = 0.71073 Å
Hall symbol: P 2c -2ac	Cell parameters from 2422 reflections
*a* = 15.445 (3) Å	θ = 2.3–25.1º
*b* = 9.753 (2) Å	µ = 0.07 mm^−^^1^
*c* = 11.411 (2) Å	*T* = 293 (2) K
*V* = 1718.9 (6) Å^3^	Needle, colorless
*Z* = 4	0.24 × 0.14 × 0.08 mm

### Data collection

**Table:**

Rigaku R-AXIS RAPID IP area-detector diffractometer	1607 independent reflections
Radiation source: Rotating Anode	1291 reflections with *I* > 2σ(*I*)
Monochromator: graphite	*R*_int_ = 0.038
*T* = 293(2) K	θ_max_ = 25.0º
ω oscillation scans	θ_min_ = 3.1º
Absorption correction: multi-scan(ABSCOR; Higashi, 1995)	*h* = −18→18
*T*_min_ = 0.982, *T*_max_ = 0.994	*k* = −11→11
12952 measured reflections	*l* = −12→13

### Refinement

**Table:**

Refinement on *F*^2^	Secondary atom site location: difference Fourier map
Least-squares matrix: full	Hydrogen site location: inferred from neighbouring sites
*R*[*F*^2^ > 2σ(*F*^2^)] = 0.041	H-atom parameters constrained
*wR*(*F*^2^) = 0.114	*w* = 1/[σ^2^(*F*_o_^2^) + (0.0488*P*)^2^ + 0.3923*P*] where *P* = (*F*_o_^2^ + 2*F*_c_^2^)/3
*S* = 1.13	(Δ/σ)_max_ < 0.001
1607 reflections	Δρ_max_ = 0.17 e Å^−^^3^
218 parameters	Δρ_min_ = −0.15 e Å^−^^3^
1 restraint	Extinction correction: SHELXTL (Sheldrick, 2008), Fc^*^=kFc[1+0.001xFc^2^λ^3^/sin(2θ)]^-1/4^
Primary atom site location: structure-invariant direct methods	Extinction coefficient: 0.028 (3)

### Special details

**Table:**

Geometry. All e.s.d.'s (except the e.s.d. in the dihedral angle between two l.s. planes) are estimated using the full covariance matrix. The cell e.s.d.'s are taken into account individually in the estimation of e.s.d.'s in distances, angles and torsion angles; correlations between e.s.d.'s in cell parameters are only used when they are defined by crystal symmetry. An approximate (isotropic) treatment of cell e.s.d.'s is used for estimating e.s.d.'s involving l.s. planes.
Refinement. Refinement of *F*^2^ against ALL reflections. The weighted *R*-factor *wR* and goodness of fit *S* are based on *F*^2^, conventional *R*-factors *R* are based on *F*, with *F* set to zero for negative *F*^2^. The threshold expression of *F*^2^ > σ(*F*^2^) is used only for calculating *R*-factors(gt) *etc*. and is not relevant to the choice of reflections for refinement. *R*-factors based on *F*^2^ are statistically about twice as large as those based on *F*, and *R*- factors based on ALL data will be even larger.

### Fractional atomic coordinates and isotropic or equivalent isotropic displacement parameters (Å^2^)

**Table:**

	*x*	*y*	*z*	*U*_iso_*/*U*_eq_	
N4	0.12629 (16)	0.3089 (3)	0.5140 (3)	0.0547 (7)	
N3	0.19406 (17)	0.5004 (3)	0.5604 (3)	0.0552 (7)	
C10	0.19739 (18)	0.3857 (3)	0.4962 (3)	0.0486 (7)	
C15	0.2983 (2)	0.6672 (3)	0.6431 (3)	0.0554 (8)	
C11	0.2649 (2)	0.3501 (3)	0.4181 (3)	0.0563 (8)	
C6	0.0325 (2)	0.1189 (4)	0.4561 (4)	0.0645 (10)	
C13	0.2496 (2)	0.2696 (4)	0.3185 (4)	0.0604 (9)	
C8	0.0718 (2)	0.3705 (4)	0.6057 (4)	0.0651 (10)	
H8A	0.0726	0.3157	0.6766	0.078*	
H8B	0.0125	0.3805	0.5793	0.078*	
C14	0.2426 (3)	0.6261 (3)	0.5409 (3)	0.0628 (9)	
H14A	0.2022	0.6997	0.5242	0.075*	
H14B	0.2791	0.6142	0.4725	0.075*	
C12	0.3498 (2)	0.3964 (5)	0.4360 (4)	0.0730 (11)	
N2	0.2371 (3)	0.2064 (3)	0.2348 (3)	0.0805 (10)	
C7	0.1204 (2)	0.1601 (3)	0.5002 (4)	0.0633 (9)	
H7A	0.1313	0.1161	0.5751	0.076*	
H7B	0.1643	0.1293	0.4454	0.076*	
C16	0.3438 (2)	0.5709 (4)	0.7070 (3)	0.0606 (9)	
H16A	0.3370	0.4782	0.6903	0.073*	
C18	0.4101 (3)	0.7463 (5)	0.8220 (4)	0.0808 (12)	
H18A	0.4479	0.7731	0.8811	0.097*	
C19	0.3648 (3)	0.8422 (5)	0.7607 (4)	0.0882 (14)	
H19A	0.3718	0.9345	0.7788	0.106*	
C9	0.1137 (2)	0.5087 (4)	0.6255 (4)	0.0648 (9)	
H9A	0.0774	0.5820	0.5958	0.078*	
H9B	0.1247	0.5241	0.7081	0.078*	
C20	0.3079 (3)	0.8040 (4)	0.6708 (4)	0.0746 (11)	
H20A	0.2768	0.8704	0.6302	0.089*	
C17	0.3997 (2)	0.6111 (5)	0.7963 (3)	0.0688 (10)	
H17A	0.4301	0.5454	0.8386	0.083*	
C4	−0.0941 (3)	−0.0245 (5)	0.4651 (7)	0.109 (2)	
H4A	−0.1243	−0.0959	0.5002	0.130*	
N1	0.4199 (2)	0.4303 (5)	0.4518 (4)	0.1066 (15)	
C5	−0.0132 (3)	0.0158 (4)	0.5091 (5)	0.0864 (14)	
H5A	0.0095	−0.0279	0.5747	0.104*	
C1	−0.0043 (3)	0.1819 (5)	0.3600 (5)	0.0978 (15)	
H1A	0.0257	0.2515	0.3219	0.117*	
C2	−0.0843 (4)	0.1437 (6)	0.3197 (7)	0.124 (2)	
H2A	−0.1085	0.1892	0.2561	0.149*	
C3	−0.1278 (4)	0.0414 (6)	0.3712 (8)	0.123 (2)	
H3A	−0.1817	0.0155	0.3423	0.147*	

### Atomic displacement parameters (Å^2^)

**Table:**

	*U*^11^	*U*^22^	*U*^33^	*U*^12^	*U*^13^	*U*^23^
N4	0.0400 (13)	0.0606 (15)	0.0636 (18)	−0.0039 (12)	0.0008 (14)	−0.0105 (14)
N3	0.0491 (15)	0.0576 (15)	0.0590 (16)	−0.0077 (12)	−0.0039 (13)	−0.0092 (15)
C10	0.0395 (15)	0.0585 (17)	0.0477 (18)	0.0002 (13)	−0.0019 (15)	0.0002 (16)
C15	0.0533 (18)	0.0564 (18)	0.056 (2)	−0.0081 (15)	−0.0014 (17)	−0.0045 (17)
C11	0.0457 (18)	0.069 (2)	0.054 (2)	−0.0041 (15)	0.0006 (16)	−0.0024 (18)
C6	0.059 (2)	0.0561 (19)	0.078 (3)	−0.0091 (17)	0.005 (2)	−0.0164 (19)
C13	0.0567 (19)	0.067 (2)	0.057 (2)	0.0062 (17)	0.0012 (18)	−0.001 (2)
C8	0.0442 (18)	0.083 (2)	0.068 (2)	−0.0025 (17)	0.0070 (17)	−0.014 (2)
C14	0.065 (2)	0.0590 (18)	0.065 (2)	−0.0084 (18)	−0.0123 (18)	0.0010 (19)
C12	0.055 (2)	0.100 (3)	0.064 (2)	−0.010 (2)	0.0075 (19)	−0.012 (2)
N2	0.093 (3)	0.082 (2)	0.067 (2)	0.008 (2)	0.006 (2)	−0.011 (2)
C7	0.0579 (19)	0.0544 (18)	0.078 (2)	−0.0037 (15)	−0.001 (2)	−0.006 (2)
C16	0.056 (2)	0.069 (2)	0.056 (2)	−0.0103 (18)	0.0020 (17)	−0.0012 (19)
C18	0.065 (2)	0.113 (3)	0.065 (2)	−0.026 (2)	0.000 (2)	−0.017 (3)
C19	0.090 (3)	0.083 (3)	0.092 (3)	−0.030 (3)	0.012 (3)	−0.032 (3)
C9	0.057 (2)	0.075 (2)	0.063 (2)	−0.0002 (18)	0.0069 (19)	−0.0148 (19)
C20	0.079 (3)	0.060 (2)	0.084 (3)	−0.0100 (19)	0.003 (2)	−0.012 (2)
C17	0.0524 (19)	0.100 (3)	0.053 (2)	−0.0134 (19)	−0.0046 (17)	−0.002 (2)
C4	0.075 (3)	0.074 (3)	0.177 (6)	−0.023 (2)	0.016 (4)	−0.025 (4)
N1	0.054 (2)	0.153 (4)	0.113 (3)	−0.020 (2)	0.015 (2)	−0.039 (3)
C5	0.076 (3)	0.068 (2)	0.115 (4)	−0.015 (2)	0.016 (3)	−0.011 (3)
C1	0.096 (3)	0.094 (3)	0.103 (4)	−0.023 (3)	−0.034 (3)	0.004 (3)
C2	0.109 (4)	0.110 (4)	0.153 (6)	−0.019 (3)	−0.066 (4)	−0.014 (4)
C3	0.086 (4)	0.089 (4)	0.194 (7)	−0.012 (3)	−0.029 (4)	−0.039 (4)

### Geometric parameters (Å, °)

**Table:**

N4—C10	1.345 (4)	C7—H7A	0.9700
N4—C7	1.463 (4)	C7—H7B	0.9700
N4—C8	1.470 (4)	C16—C17	1.392 (5)
N3—C10	1.339 (4)	C16—H16A	0.9300
N3—C9	1.448 (4)	C18—C17	1.360 (6)
N3—C14	1.453 (4)	C18—C19	1.361 (7)
C10—C11	1.415 (5)	C18—H18A	0.9300
C15—C20	1.379 (5)	C19—C20	1.401 (6)
C15—C16	1.381 (5)	C19—H19A	0.9300
C15—C14	1.504 (5)	C9—H9A	0.9700
C11—C12	1.402 (5)	C9—H9B	0.9700
C11—C13	1.402 (5)	C20—H20A	0.9300
C6—C5	1.370 (5)	C17—H17A	0.9300
C6—C1	1.380 (6)	C4—C3	1.354 (9)
C6—C7	1.503 (5)	C4—C5	1.402 (7)
C13—N2	1.153 (5)	C4—H4A	0.9300
C8—C9	1.512 (5)	C5—H5A	0.9300
C8—H8A	0.9700	C1—C2	1.368 (6)
C8—H8B	0.9700	C1—H1A	0.9300
C14—H14A	0.9700	C2—C3	1.339 (9)
C14—H14B	0.9700	C2—H2A	0.9300
C12—N1	1.146 (5)	C3—H3A	0.9300
			
C10—N4—C7	125.9 (3)	H7A—C7—H7B	108.0
C10—N4—C8	110.3 (3)	C15—C16—C17	120.6 (4)
C7—N4—C8	116.5 (3)	C15—C16—H16A	119.7
C10—N3—C9	111.1 (3)	C17—C16—H16A	119.7
C10—N3—C14	127.0 (3)	C17—C18—C19	119.7 (4)
C9—N3—C14	118.3 (3)	C17—C18—H18A	120.1
N3—C10—N4	110.6 (3)	C19—C18—H18A	120.1
N3—C10—C11	125.3 (3)	C18—C19—C20	121.0 (4)
N4—C10—C11	124.1 (3)	C18—C19—H19A	119.5
C20—C15—C16	118.9 (4)	C20—C19—H19A	119.5
C20—C15—C14	119.8 (4)	N3—C9—C8	103.9 (3)
C16—C15—C14	121.3 (3)	N3—C9—H9A	111.0
C12—C11—C13	117.2 (3)	C8—C9—H9A	111.0
C12—C11—C10	121.3 (3)	N3—C9—H9B	111.0
C13—C11—C10	121.6 (3)	C8—C9—H9B	111.0
C5—C6—C1	117.7 (4)	H9A—C9—H9B	109.0
C5—C6—C7	120.9 (4)	C15—C20—C19	119.5 (4)
C1—C6—C7	121.3 (4)	C15—C20—H20A	120.3
N2—C13—C11	178.3 (4)	C19—C20—H20A	120.3
N4—C8—C9	103.0 (3)	C18—C17—C16	120.3 (4)
N4—C8—H8A	111.2	C18—C17—H17A	119.9
C9—C8—H8A	111.2	C16—C17—H17A	119.9
N4—C8—H8B	111.2	C3—C4—C5	119.6 (5)
C9—C8—H8B	111.2	C3—C4—H4A	120.2
H8A—C8—H8B	109.1	C5—C4—H4A	120.2
N3—C14—C15	113.7 (3)	C6—C5—C4	120.4 (5)
N3—C14—H14A	108.8	C6—C5—H5A	119.8
C15—C14—H14A	108.8	C4—C5—H5A	119.8
N3—C14—H14B	108.8	C2—C1—C6	121.2 (5)
C15—C14—H14B	108.8	C2—C1—H1A	119.4
H14A—C14—H14B	107.7	C6—C1—H1A	119.4
N1—C12—C11	177.9 (6)	C3—C2—C1	120.6 (7)
N4—C7—C6	110.9 (3)	C3—C2—H2A	119.7
N4—C7—H7A	109.5	C1—C2—H2A	119.7
C6—C7—H7A	109.5	C2—C3—C4	120.5 (6)
N4—C7—H7B	109.5	C2—C3—H3A	119.8
C6—C7—H7B	109.5	C4—C3—H3A	119.8
			
C9—N3—C10—N4	−2.2 (4)	C1—C6—C7—N4	49.6 (5)
C14—N3—C10—N4	−159.9 (3)	C20—C15—C16—C17	−1.3 (5)
C9—N3—C10—C11	176.5 (3)	C14—C15—C16—C17	175.6 (3)
C14—N3—C10—C11	18.8 (5)	C17—C18—C19—C20	−0.4 (7)
C7—N4—C10—N3	−153.8 (3)	C10—N3—C9—C8	7.8 (4)
C8—N4—C10—N3	−4.8 (4)	C14—N3—C9—C8	167.7 (3)
C7—N4—C10—C11	27.5 (5)	N4—C8—C9—N3	−9.8 (4)
C8—N4—C10—C11	176.5 (3)	C16—C15—C20—C19	1.5 (6)
N3—C10—C11—C12	28.6 (5)	C14—C15—C20—C19	−175.4 (4)
N4—C10—C11—C12	−152.9 (4)	C18—C19—C20—C15	−0.7 (7)
N3—C10—C11—C13	−150.1 (3)	C19—C18—C17—C16	0.7 (6)
N4—C10—C11—C13	28.4 (5)	C15—C16—C17—C18	0.2 (6)
C10—N4—C8—C9	9.3 (4)	C1—C6—C5—C4	0.9 (6)
C7—N4—C8—C9	161.4 (3)	C7—C6—C5—C4	−178.2 (4)
C10—N3—C14—C15	−121.6 (4)	C3—C4—C5—C6	−1.5 (7)
C9—N3—C14—C15	82.0 (4)	C5—C6—C1—C2	0.7 (7)
C20—C15—C14—N3	−143.2 (3)	C7—C6—C1—C2	179.7 (5)
C16—C15—C14—N3	39.9 (5)	C6—C1—C2—C3	−1.7 (9)
C10—N4—C7—C6	−145.9 (3)	C1—C2—C3—C4	1.0 (10)
C8—N4—C7—C6	66.8 (4)	C5—C4—C3—C2	0.5 (9)
C5—C6—C7—N4	−131.3 (4)		
